# Impact of Uniaxial Static Strain on Myoblast Differentiation in Collagen-Coated PCL Microfilament Scaffolds: Role of Onset Time of Mechanical Stimulation

**DOI:** 10.3390/bioengineering11090919

**Published:** 2024-09-13

**Authors:** María Laura Espinoza-Álvarez, Laura Rojas-Rojas, Johan Morales-Sánchez, Teodolito Guillén-Girón

**Affiliations:** 1 Materials Science and Engineering School, Instituto Tecnológico de Costa Rica, Cartago 30101, Costa Rica; laurarojas@itcr.ac.cr (L.R.-R.); tguillen@itcr.ac.cr (T.G.-G.); 2 Tissue Engineering Laboratory, Biotechnology Research Center, Instituto Tecnológico de Costa Rica, Cartago 30101, Costa Rica; jmorales@itcr.ac.cr; 3 Physics School, Instituto Tecnológico de Costa Rica, Cartago 30101, Costa Rica; 4 PhD Program in Sciences, Universidad de La Frontera, Temuco 4811230, Chile

**Keywords:** C2C12 cell line, polycaprolactone, bioreactor, myogenic markers, alignment

## Abstract

Tissue engineering endeavors to create in vitro constructs that replicate the properties of native tissue, such as skeletal muscle. This study investigated the use of mechanical stimulation to promote myogenic differentiation and enhance the functionality of bioengineered tissues. Specifically, it aimed to facilitate the differentiation of myoblasts within a three-dimensional scaffold using a defined pattern of mechanical stimulation. C2C12 cells were cultured on a collagen-coated PCL microfilament scaffold and subjected to 24 h of uniaxial static strain using a biomechanical stimulation system. Two onset times of stimulation, 72 h and 120 h post-seeding, were evaluated. Cell proliferation, myogenic marker expression, and alterations in cell morphology and orientation were assessed. Results indicate that static strain on the scaffold promoted myoblast differentiation, evidenced by morphological and molecular changes. Notably, strain initiated at 72 h induced an early differentiation stage marked by MyoD expression, whereas stimulation beginning at 120 h led to a mid-stage differentiation characterized by the co-expression of MyoD and Myogenin, culminating in myotube formation. These results highlight the critical influence of myoblast maturity at the time of strain application on the differentiation outcome. This study provides insights that could guide the optimization of mechanical stimulation protocols in tissue engineering applications.

## 1. Introduction

Skeletal muscle stands out for its high level of plasticity, which allows it to respond and adapt to different physiological demands and stimuli such as contractile activity, mechanical loading, nutritional status, and hypoxia [1]. Based on its plasticity, the tissue is able to repair in response to a minor injury, such as strain generated by exercise; however, this capacity is compromised by genetic myopathies, aging, and the loss of large amounts of muscle mass and contractile proteins (volumetric muscle loss) [2,3].

Tissue engineering aims to develop in vitro constructs that mimic the anatomical and functional characteristics of native tissue [4]. These bioengineered tissues have a wide range of applications in clinical and biomedical settings that include serving as study models, facilitating drug screening, providing therapeutic transplants, and supporting basic research endeavors [2]. Nevertheless, to develop a biomimetic tissue it is necessary to recreate its microenvironment, including the cells that constitute the tissue, the substrate in which they anchor, and the biological and external signals they perceive [1,5].

Myoblasts are myogenic progenitor cells that exit the cell cycle after proliferation in order to fuse and differentiate into multinucleated myotubes, subsequently maturing into muscle fibers [6]. This developmental process is controlled by a complex gene regulatory network [7]. Different transcription factors have been associated with markers of myogenesis progression. For example, the myogenic regulatory factors (MRFs), composed of Myf5, MyoD, Myogenin and MRF4, are spatially and temporally activated in order to establish the skeletal muscle phenotype [8,9]. In general, Myf5 and MyoD are determinants of myogenesis, as they guide precursor cells to initiate skeletal muscle development. Myogenin acts together with MyoD and MRF4 to activate and promote the terminal differentiation and expression of myotube-specific genes [10,11].

Scaffolds provide a temporal structural support for cells to attach to, proliferate, and eventually produce their own extracellular matrix for tissue formation [12,13]. Polycaprolactone (PCL) is a biocompatible biomaterial currently used for scaffold development, since it is easily processed and molded into different shapes, its biodegradability and mechanical properties can be modified according to its intended use, and it is susceptible to surface modifications [4,14]. Collagen may be utilized for scaffold coating to enhance surface biological interaction. Collagen has the RGD (Arg-Gly-Asp) cell recognition motif, which promotes cell adhesion and proliferation, facilitating tissue development [15,16,17]. 

External cues like mechanical stimuli affect cell fate and regeneration in skeletal muscle [18]. Strain regimes can be applied during in vitro culture to promote myogenic differentiation and maturity, and to enhance tissue functionality [19]. Evidence has shown that strain and scaffold topography trigger myoblast alignment [20], which is crucial for fusion into myotubes [21]. Nevertheless, cells respond differently depending on the type of strain regimen (i.e., static or cyclic, uniaxial or biaxial) and the parameters applied (i.e., onset time, duration, frequency, and amplitude) [22,23]. Although no consensus has been reached on the optimal regimen, static strains have been utilized to mimic in vivo embryonic development, where the muscles are subjected to strain due to bone elongation [23]. 

Investigating the optimal conditions for applying strain is a key step in replicating the physiological myogenic characteristics in engineered tissues. The onset time or duration of mechanical stimulation can lead to varying results. Therefore, the same loading pattern can be applied to a cell-seeded scaffold at different onset times to evaluate whether the stages of tissue development or maturation could potentially result in varying myogenic outcomes [23].

The main aim of this study was to promote the differentiation of myoblasts from the C2C12 cell line seeded on a three-dimensional polymeric scaffold by means of a mechanical stimulation pattern. C2C12 cells were seeded in a collagen-coated PCL microfilament scaffold and subjected to 24 h of uniaxial static strain in a custom-made biomechanical stimulation system. The initiation timings of mechanical stimulation at 72 h and 120 h were assessed. The outcomes of the experiments demonstrated that the mechanical stimulation protocol facilitated the differentiation of C2C12 cells. Moreover, a strain onset time of 120 h enhanced myogenic outcomes such as gene expression levels and myotube formation.

## 2. Materials and Methods

### 2.1. Materials and Reagents

Polycaprolactone Mn 80,000 g/mol pellets (Sigma-Aldrich, Milwaukee, WI, USA, Catalog No. 440744) were used in the scaffold fabrication and Collagen Coating Solution (Cell Applications Inc., San Diego, CA, USA, Catalog No. 125-50) was utilized for coating.

A C2C12 mouse myoblast cell line (CRL 1772^TM^, American Type Culture Collection, ATCC, Manassas, VA, USA) was used throughout the investigation. Cells were grown in a growth medium, which consisted of Dulbecco’s Modified Eagle Medium (DMEM) (Gibco^TM^, Grand Island, NY, USA, Catalog No. 11995-065), supplemented with 1% penicillin-streptomycin (P/S) (Gibco^TM^, Catalog No. 15140-122) and 10% fetal bovine serum (FBS) (Gibco^TM^, Catalog No. 10437-028). Trypsin-EDTA 0.05% (Gibco^TM^, Catalog No. 25200-072) and phosphate-buffered saline (PBS) (Sigma-Aldrich, Catalog No. P4417-100TAB) were used for subculturing cells.

Alamar Blue^TM^ Cell Viability Reagent (Invitrogen by Thermo Fisher Scientific, Eugene, OR, USA, Catalog No. DAL1100) was used for cell proliferation assays. The following reagents were used for immunofluorescence staining procedure: paraformaldehyde (PFA) (Thermo Fisher Scientific^TM^, Catalog No. O4042-500), Triton X-100 (Sigma-Aldrich, Catalog No. T X-100), bovine serum albumin (BSA) (Thermo Fisher Scientific^TM^, Catalog No. BP9700-100), 4′, 6-diamidino-2-phenylindole (DAPI) (Santa Cruz Biotechnology, Dallas, TX, USA, Catalog No. sc-3598, CAS No. 28718-90-3), Alexa Fluor 488 Phalloidin (Invitrogen by Thermo Fisher Scientific, Catalog No. A-12370), Anti-MyoD antibody (Invitrogen by Thermo Fisher Scientific, Catalog No. PA5-23078), Anti-Myogenin antibody (abcam, Waltham, MA, USA, Catalog No. Ab1835), Goat anti-rabbit Alexa Fluor 594 (Invitrogen by Thermo Fisher Scientific, Catalog No. A-11012), and Goat anti-mouse Alexa Fluor 594 (Invitrogen by Thermo Fisher Scientific, Catalog No. A-11005). 

All reagents were of analytical grade quality and were used according to the manufacturer’s instructions. 

### 2.2. Cell Culture

The C2C12 cells were plated in standard flasks with growth medium. The flask was kept in a humidified incubator set at 5% CO_2_ and 37 °C (standard conditions). The medium was changed every 2 days. Cells were subcultured at 60–70% confluence using Trypsin-EDTA 0.05%. Cell cultures with passages of 25–42 were used throughout the research. 

### 2.3. Manufacture of Scaffolds and Cell Seeding

Polycaprolactone pellets were processed into a filament using a Filabot Ex2 extruder (Filabot, Barre, VT, USA), operating at 80 °C, equipped with a 1 mm diameter die. The resulting filament was then wound onto a spool using a spooler. Subsequently, the filament underwent stretching until the plastic yield point was reached, corresponding to a strain (ε) of approximately 97%.

The microfilaments were arranged as a bundle of 61 aligned microfilaments in a 360 L stainless steel grip. The grip was used as an alignment and support mechanism for the microfilaments. In addition, the grip was designed to transfer mechanical loads to the microfilaments by connecting to a universal testing machine. Further information about the manufacturing process and material characterization can be found in [24,25].

The grip with the arranged microfilaments was sterilized using 25 kGy of gamma rays, in an Ob-Servo Ignis with a Co-60 source gamma irradiator. The sterile microfilaments were coated by depositing 600 µL of the Collagen Coating Solution on the surface of the scaffold and incubating at standard conditions for 30 min. The microfilaments were washed three times with 1 mL of sterile PBS after the incubation period. 

Approximately 3 × 10^5^ cells were seeded in the microfilaments. To promote cell adhesion, the cells were directly seeded on the surface of the scaffold by adding 300 µL of cell suspension. After 2 h of incubation under standard conditions, 3 mL of growth medium was slowly added to prevent the cells from dispersing.

### 2.4. Biomechanical Stimulation System Setup 

The biomechanical stimulation was performed using a bioreactor, a water bath, and a measurement system for environmental parameter control. The bioreactor facilitated a closed and sterile environment optimal for cell growth. The water bath maintained a constant temperature of 37 ± 1 °C, essential for cellular activities. The environmental measurement system regulated the bioreactor atmosphere by supplying 5% CO_2_ and sustaining high humidity levels via a micropump.

**Figure 1:**
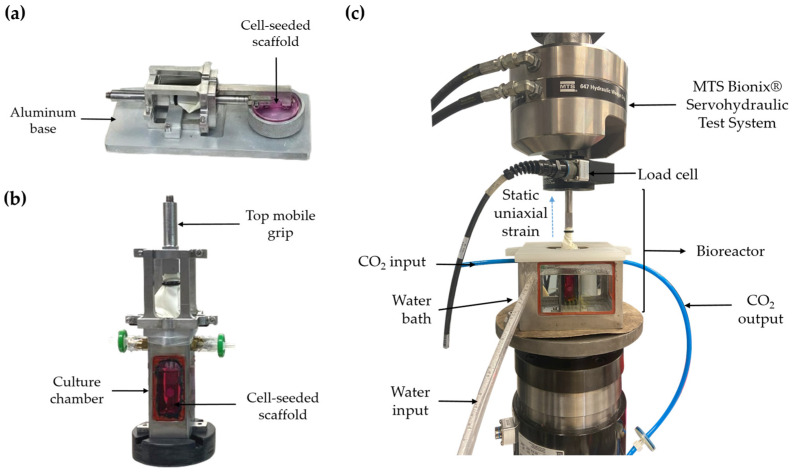
(**a**) Diagram of the horizontal microfilament grip. (**b**) Diagram of the bioreactor. (**c**) Diagram of the biomechanical stimulation system.

Following 72 or 120 h of cell seeding—dependent on the mechanical stimulation onset time—the bioreactor was assembled. The microfilament grip, initially in a horizontal orientation on an aluminum base (as shown in Figure 1a), was repositioned vertically within a stainless steel and glass culture chamber containing 25 mL of growth medium (Figure 1b). Subsequently, two conduits were connected to the bioreactor ports to facilitate the inflow and outflow of CO_2_. The bioreactor was then placed in the water bath, and, finally, the top grip was secured to a universal testing machine (Figure 1c).

As a control, a cell-seeded scaffold was maintained horizontally in its aluminum base within a commercial incubator for the entirety of the experiment.

### 2.5. Mechanical Stimulation of the Cell-Seeded Scaffold

The cell-seeded scaffold was subjected to static strain to evaluate the effect on cell proliferation, alignment, and the expression of myogenic markers associated with differentiation progress. Different onset times were tested, where strain was applied either at day 3 (72 h) or day 5 (120 h) after cell seeding. Table 1 shows the experimental timeline of the mechanical stimulation tests.

**Figure 2:**
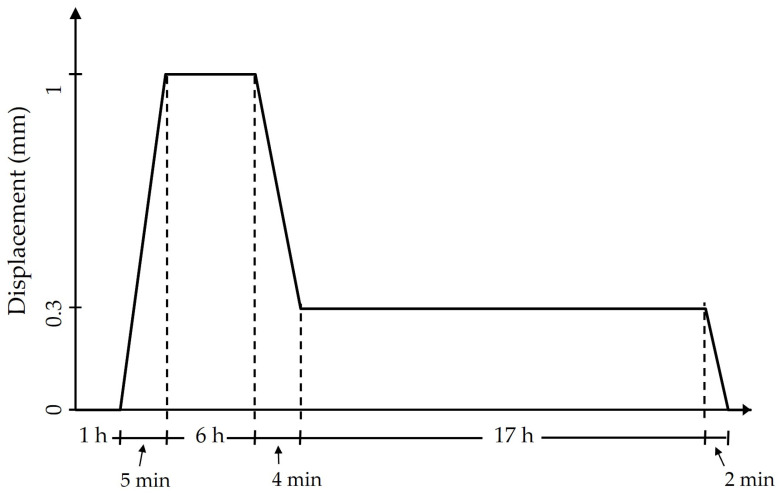
Loading pattern used on the cell-seeded scaffold.

The mechanical tensile properties of the microfilaments were assessed using a servohydraulic test system (Model 810, MTS Systems Corporation, Eden Prairie, MN, USA), equipped with a 250 N load cell. Each test involved monotonically loading the microfilament according to the pattern depicted in Figure 2. The static strain pattern for stimulation was adopted from Heher et al. [23] and informed by results from previous experiments [24], with onset times determined based on preliminary findings. The initial configuration of the microfilaments was 22 mm in length. Previous studies have been conducted to examine the mechanical properties of these scaffolds [25] in which 1 mm and 0.3 mm displacement remain within the elastic regime of the sample.

The loading pattern proceeded as follows: The cell-seeded microfilaments were first allowed to acclimate to the environment for one hour while remaining stationary. The sample was then stretched at a rate of 0.2 mm/min until it reached an extension of 1 mm. This extended state was maintained for six hours, after which the sample was unloaded at a rate of 0.18 mm/min until it reached 0.3 mm, where it remained for 17 h, completing a 24 h cycle. Before removing the sample from the equipment, it was returned to its initial length of 0 mm within two minutes.

Stress was calculated by dividing the measured force by the combined cross-sectional area of 61 microfilaments, each with a diameter of 90 µm.

### 2.6. Cell Proliferation Analysis

Cell proliferation was assessed with Alamar Blue^TM^ Cell Viability Reagent (Invitrogen by Thermo Fisher Scientific, Eugene, OR, USA, Catalog No. DAL1100) immediately prior to the initiation of mechanical stimulation, and 24 h after. This procedure was performed by placing the microfilaments in a horizontal position in the base-holder and adding 3 mL of growth medium with 10% Alamar Blue^TM^ to the cell-seeded scaffold. After 1 h of incubation at standard conditions, 200 µL of the reduced compound were transferred to a 96-well flat bottom microplate. Relative fluorescence units (RFU) were measured at a 544/590 nm (Ex/Em) wavelength using a Fluostar Optima (BMG LABTECH, San Diego, CT, USA). The percentage of cell proliferation was calculated by considering the RFU of the strained and positive control scaffold at 72/120 h as 100% proliferation. Equations (1) and (2) were followed to calculate the cell proliferation percentage.

(1) $$Cell\;proliferation\left(\%\right)=\left(\frac{Strained\;scaffold\;RFU}{Strained\;scaffold\;RFU\;at\;72/120\;h}\right)\times 100$$

(2) $$Cell\;proliferation\left(\%\right)=\left(\frac{Positive\;control\;RFU}{Positive\;control\;RFU\;at\;72/120\;h}\right)\times 100$$

### 2.7. Immunostaining

After mechanical stimulation and measurement of proliferation, the cell-seeded scaffolds were washed with PBS and fixed with PFA 4% for 20 min at room temperature. PFA was removed, and the cells were washed three times with PBS. Permeabilization solution (0.5% Triton X-100 in PBS) was added to the fixed cells for 10 min. After the permeabilization solution was removed, PBS was used to wash the scaffold. The cells were treated with blocking solution (1% BSA in PBS) for 30 min, and then they were washed with washing solution (0.01% Triton X-100 in PBS) for 1 min. Primary antibodies Anti-MyoD (rabbit polyclonal IgG) 1:100 in washing solution and Anti-Myogenin (mouse monoclonal IgG1) 1:50 in washing solution were added and incubated overnight at 4 °C. The primary antibodies were then removed, and the cells were washed twice for 5 min with washing solution. The samples were exposed for 1 h to the secondary antibodies Goat anti-rabbit Alexa Fluor 594 and Goat anti-mouse Alexa Fluor 594, both diluted at a ratio of 1:300 in washing solution. Finally, the cells were washed twice with the washing solution for 30 s. 

The actin filaments of the cytoskeleton of cells were stained with Alexa Fluor 488 Phalloidin, 1:40 in PBS for 30 min. DAPI, 1:1000 in PBS, was used to counterstain cell nuclei. Different samples were used for MyoD and Myogenin marker analysis, since both corresponding secondary antibodies had an excitation/emission of 590/618 nm. The microfilaments with the stained cells were analyzed in a Leica Dmi8 inverted fluorescence microscope. 

### 2.8. Image Processing

Images obtained from immunostaining were processed using ImageJ software version 1.54f. Background subtraction and channel merging were performed to enhance image clarity. Brightness and contrast adjustments were made individually for each color channel to optimize visibility.

Nuclei alignment was quantified using the directionality tool within the software, which generates a histogram depicting the orientation of structures within a specified direction. DAPI-stained images were specifically used for this analysis. Each histogram was constructed by averaging the directionality data extracted from micrographs of three separate experiments involving both strained and control scaffolds. To standardize the orientation across all samples, micrographs were adjusted with a 0° tilt rotation, ensuring that the microfilaments were horizontally aligned. This adjustment typically resulted in a single directionality peak at 0°. The proportion of nuclei oriented within a −5° to 5° range was then calculated against the total number of nuclei to determine the percentage of alignment. The Fourier components method was employed for a more detailed directionality analysis.

### 2.9. Statistical Analysis

Statistical analyses were conducted using Minitab18 software. Data from cell proliferation and alignment studies are presented as the mean ± standard deviation, based on triplicate samples. The Anderson-Darling test was used to evaluate normality distribution. To identify significant differences between samples, a one-way analysis of variance (ANOVA) followed by either Tukey’s multiple comparison test or the Games–Howell test, were used depending on the homogeneity of variances. Additionally, the two-sample *t*-test and the Mann–Whitney test were used where appropriate. *p*-values of 0.05 or less were considered statistically significant.

## 3. Results

### 3.1. Application of Static Strain at 72 h of Culture Promoted an Early Differentiation Stage of Myoblasts

**Figure 3:**
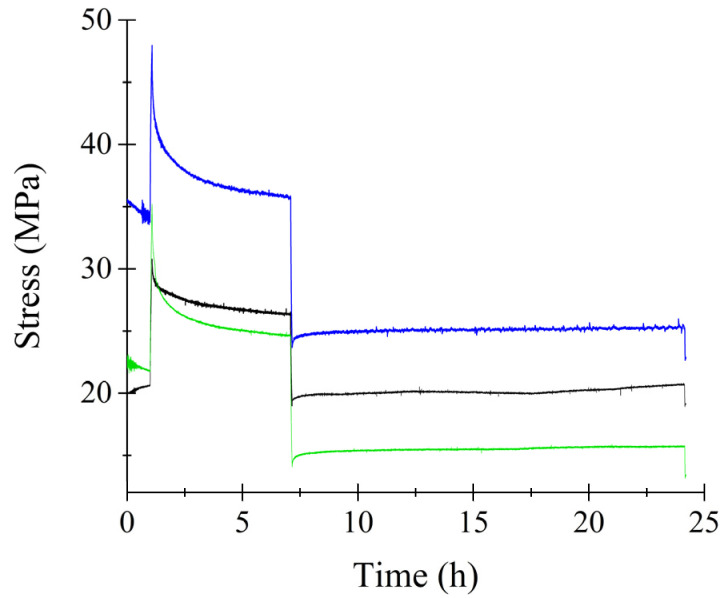
Stress–strain curve of microfilaments during mechanical stimulation. Onset time of mechanical stimulation was 72 h. Colored lines represent different replicas of the experiment.

Static uniaxial strain was applied to the cell-seeded scaffold after 72 h of cell seeding for a duration of 24 h. Figure 3 shows the resulting stress–strain curves for the triplicate study of the microfilaments. Initially, the curve shows a constant stress level. Initially stress remained constant for 1 h. Then, an increase of 10 MPa was observed, reaching the highest stress during the test. The samples remained loaded for 6 h. A sharp relaxation occurred at 7 h when displacement reached 0.3 mm. After unloading, stress remained constant for 17 h for the remainder of the test.

**Figure 4:**
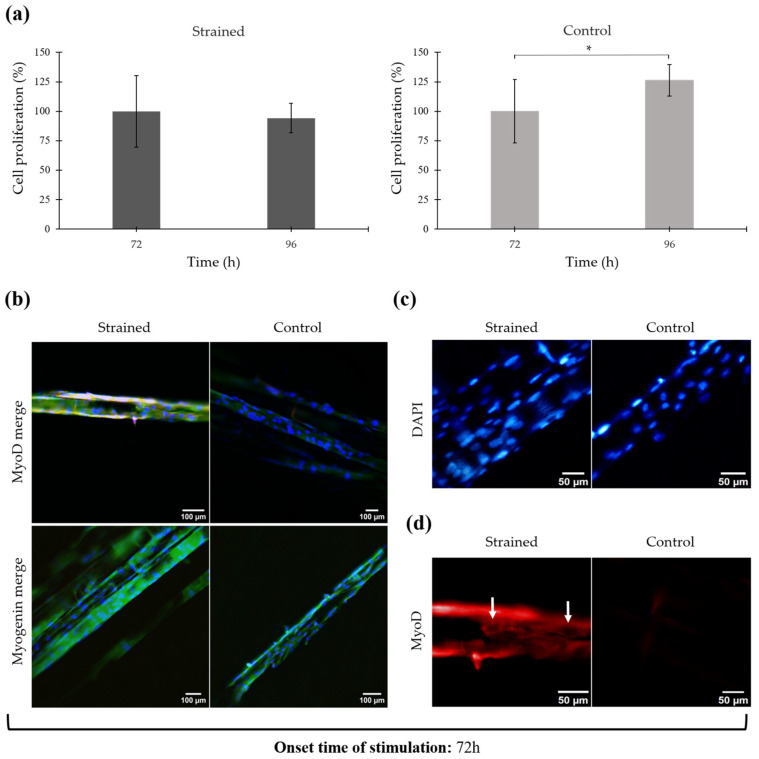
Proliferation and immunofluorescence of mechanically stimulated cells after 72 h of culture. (**a**) Cell proliferation percentage before and after mechanical stimulation. Error bars are standard deviation. * *p* ≤ 0.05. (**b**) Immunofluorescent staining after mechanical stimulation. Merge shows the overlay of DAPI (blue), Phalloidin (green), and MyoD/Myogenin (red) 20×. (**c**) Nuclei elongation and alignment after mechanical stimulation. DAPI staining (blue). Electronically zoomed-in micrograph, original magnification in microscope 20×. (**d**) Expression of MyoD myogenic marker after mechanical stimulation (red). Electronically zoomed-in micrograph, original magnification in microscope 20×.

Cell differentiation after static strain testing is shown in Figure 4. This analysis encompasses measurements of cell proliferation, expression of MyoD and Myogenin markers, and observations of changes in cell morphology and orientation, including nuclear elongation and uniaxial alignment.

Figure 4a illustrates the proliferation percentages of the cells before and after mechanical stimulation at 72 h and 96 h, respectively. It also compares these to the control cells (unstrained) at the same time points. Proliferation in the strained cells showed a slight decline from 100.00 ± 30.41% to 94.26 ± 12.55%, which was not statistically significant (*p* = 0.25). In contrast, the proliferation of control cells significantly increased from 100.00 ± 27.00% to 126.26 ± 13.24% (*p* = 0.03).

Cell proliferation evaluation indicates that the cells retained viability after mechanical stimulation, suggesting the loading pattern did not induce detrimental effects. Despite maintaining viability, as depicted in Figure 4a, there was a slight decrease in the proliferation percentage among the mechanically stimulated cells. These observations imply that while the cells remained alive, they did not exhibit active proliferation. This could indicate a potential shift in cellular activity from proliferation towards differentiation or other cellular processes in response to mechanical cues. Further investigations are required to delineate the specific mechanisms by which mechanical strain influences cellular behavior, potentially redirecting cellular energy from proliferation to differentiation or other stress responses.

After mechanical stimulation testing, myogenic markers associated with differentiation progress were analyzed using immunofluorescence techniques. Specifically, MyoD and Myogenin markers were identified, while DAPI and Alexa Fluor 488 Phalloidin were employed to counterstain the nuclei and actin filaments of the cytoskeleton, respectively. The overlay of the fluorescent staining for the strained and control cell-seeded scaffold is shown in Figure 4b, in which the nuclei are observed in blue, actin filaments in green, and MyoD/Myogenin in red. (For individual micrographs of each stain, refer to the Supplementary Materials, Figure S1).

Observations reveal that numerous nuclei are present on the surface of both the mechanically stimulated and control scaffolds, with most nuclei exhibiting an elongated morphology. Actin filaments also appear elongated on the microfilament surface and extended along their direction. The elongation of the cells coincided with the alignment of the microfilaments, showing a parallel distribution to each other. Control samples also exhibited cell elongation and alignment; however, these features were more significant in the strained scaffold, which experienced increased strain from the active uniaxial loading (refer to Figure 4c).

Further, nuclei alignment quantification performed with ImageJ showed a higher number of nuclei with a preferred orientation on the strained sample. The strained sample showed 29.25 ± 9.44% of nuclei oriented within a −5° to 5° angle, compared to 23.00 ± 5.38% in the control sample, although this difference was not statistically significant (*p* = 0.38). (Directionality histograms can be viewed in Supplementary Materials, Figure S2a).

Regarding the expression of myogenic markers, Figure 4d highlights the presence of the MyoD marker in the strained cell-seeded scaffold, with the arrow indicating cytoplasmic MyoD, characterized by bright and strong red coloring around the cell nuclei. In contrast, Myogenin was not clearly identified in either sample, as the staining appeared very faint and possibly indicative of background staining (refer to Figure 4b).

### 3.2. Application of Static Strain at 120 h of Culture Enhanced a Middle Differentiation Stage of Myoblasts

Upon examining the effects of uniaxial strain on cell-seeded scaffolds after 72 h of static culture, an onset time of mechanical stimulation after 120 h was also examined.

**Figure 5:**
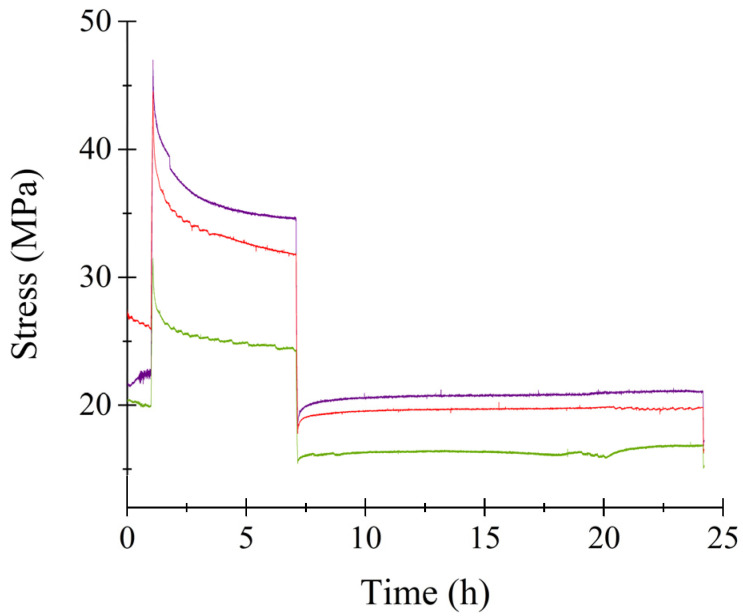
Stress–strain curve of microfilaments during mechanical stimulation. Onset time of mechanical stimulation was 120 h. Colored lines represent different replicas of the experiment.

Figure 5 displays the behavior of triplicate microfilaments under static strain initiated at 120 h of culture. The stress response observed was analogous to that depicted in Figure 3. Initially, there was an abrupt increase in stress as the microfilaments were stretched to a length of 1 mm. This was followed by a period of stress relaxation until the filament length decreased to 0.3 mm, at which stress remained constant. The cell-seeded microfilaments exhibited peak stress levels at 1 mm in length. As the length was reduced to 0.3 mm, the stress diminished to approximately 20 MPa. A notable feature of stretching these filaments is their alignment; they become highly parallel to one another, effectively mimicking the structure of biological muscle composed of parallel-loaded microfibers.

**Figure 6:**
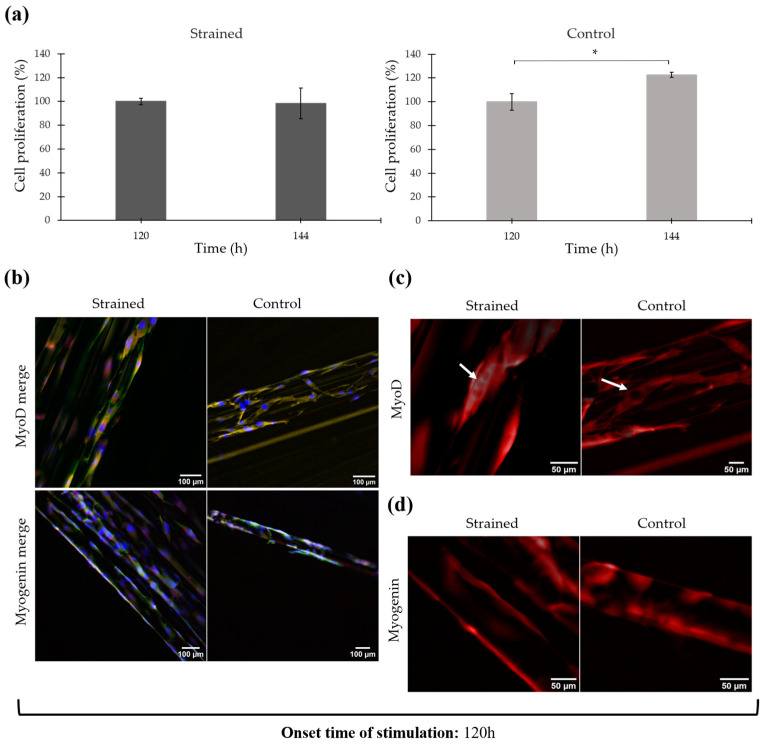
Proliferation and immunofluorescence of mechanically stimulated cells after 120 h of culture. (**a**) Cell proliferation percentage before and after mechanical stimulation. Error bars are standard deviation. * *p* ≤ 0.05. (**b**) Immunofluorescent staining after mechanical stimulation. Merge shows the overlay of DAPI (blue), Phalloidin (green), and MyoD/Myogenin (red). 20×. (**c**) Expression of MyoD myogenic marker after mechanical stimulation (red). Electronically zoomed-in micrograph, original magnification in microscope 20×. (**d**) Expression of Myogenin myogenic marker after mechanical stimulation (red). Electronically zoomed-in micrograph, original magnification in microscope 20×.

The results of cell proliferation and the expression of myogenic markers MyoD and Myogenin are illustrated in Figure 6. 

Cell proliferation percentages before and after mechanical stimulation are displayed in Figure 6a. At 120 h of cell culture, proliferation was measured as 100.00 ± 2.61%, and at 144 h, it slightly decreased to 98.45 ± 12.97%. Statistical analysis revealed no significant differences between these measurements (*p* = 0.73). In contrast, control cells exhibited significant proliferation, increasing from 100.00 ± 6.96% at 120 h to 122.56 ± 2.11% at 144 h (*p* < 0.01).

Figure 6b illustrates the immunofluorescence staining of both the strained and control cell-seeded scaffolds, showcasing an overlay of DAPI (blue), Phalloidin (green), and MyoD/Myogenin (red) markers (individual micrographs for each stain are available in the Supplementary Materials, Figure S3). Immunofluorescence staining confirmed that cells remained attached and were uniformly distributed across the scaffold after 120 h of static culture followed by 24 h of mechanical stimulation. Similar results were observed in the control sample. The cells in the strained scaffolds exhibited elongated morphology with a higher degree of alignment and orientation when compared to control cells. Quantitative analysis showed that 24.83 ± 10.60% of nuclei in the strained samples were oriented within a −5° to 5° tilt angle relative to the 0° angle of the microfilaments, while the control samples displayed 21.75 ± 15.44% alignment (directionality histograms illustrating this alignment are included in the Supplementary Materials, Figure S2b).

Concerning the expression of myogenic markers, intense red staining was observed across all samples, indicating active expression of MyoD and Myogenin in both the strained and control scaffolds (refer to Figure 6c,d). Predominantly, MyoD was localized in the cytoplasm, which is demonstrated by the absence of red coloration at the nuclei sites (highlighted by arrows in Figure 6c). However, a few nuclei did test positive for this marker. Myogenin staining was presented as a violet tint in Figure 6b, due to the overlap of DAPI and Myogenin dyes. This suggests that Myogenin is primarily localized within the nucleus, although a cytoplasmic presence was also detected. Figure 6d further confirms this, showing no unstained areas at the nuclei locations, thus verifying the presence of Myogenin-positive nuclei.

**Figure 7:**
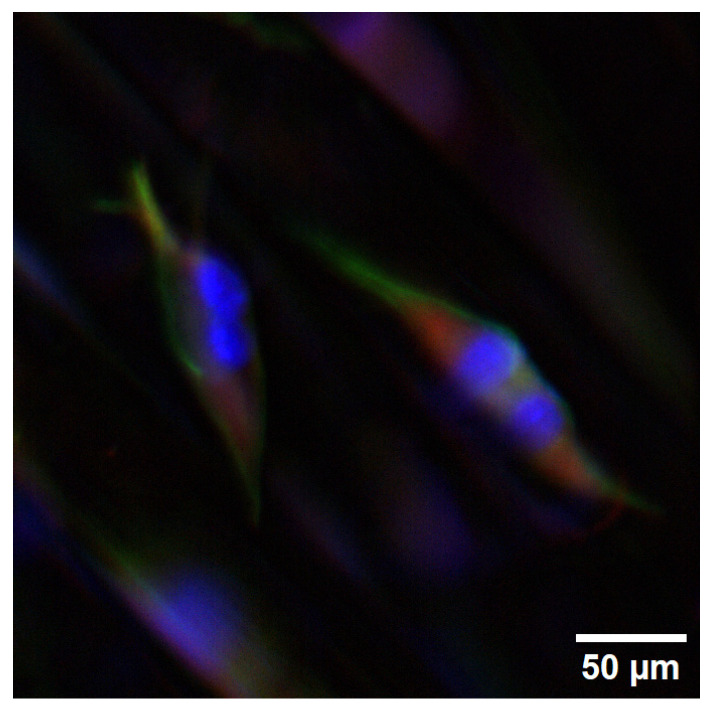
Multinucleated cells on the strained scaffold. Onset time of mechanical stimulation was 120 h. DAPI (blue), Phalloidin (green), Myogenin (red). Electronically zoomed-in micrograph, original magnification in microscope is 20×.

In the strained samples, fusion events leading to the formation of multinucleated muscle cells or myotubes were observed, illustrating the effectiveness of mechanical strain in promoting myogenic differentiation (as shown in Figure 7). In contrast to the control sample in which nuclei were single upon examination. 

## 4. Discussion

Mechanotransduction is the process through which cells sense and convert mechanical signals into biochemical responses and alterations in gene expression [26]. Cells respond to mechanical loads applied to the extracellular matrix (ECM) via integrin-mediated adhesions. These integrins are linked to the cytoskeletal filament networks, which connect directly to the nucleus, triggering membrane-signaling events and structural rearrangements within the cytoplasm and nucleus that ultimately influence gene expression [27,28]. 

As it has been reported in the literature, diverse loading patterns have been used to enhance myogenic outcomes by promoting mechanotransduction pathways. For instance, Candiani et at. [29] used unidirectional stretching and cyclic stretching. Heher et al. [20] used static strain for 6 days and Somers et al. [23] used static or cyclic strain, among other authors who have reported similar results [30,31]. In the present research, static strain was applied to the collagen-coated PCL scaffold, as it mimics the development of muscle tissue during embryogenesis, where muscle is subjected to strain as a result of skeletal growth [20].

According to Pien et al. [32], PCL is commonly used and the C2C12 cell line is chosen in more than half of the studies. While this study utilized the C2C12 myoblast cell line as a model system for investigating the effects of mechanical stimulation on myogenic differentiation, it is important to acknowledge the limitations of this model in comparison to primary muscle cells. C2C12 cells are widely used due to their robust proliferation and differentiation capabilities under controlled conditions. However, they may not fully replicate the behavior of primary myoblasts or satellite cells isolated from adult muscle tissue, which can often display a more heterogeneous population with varying proliferation and differentiation potentials [33]; these factors can influence their response to mechanical stimuli. Moreover, primary cells are more likely to maintain the native molecular, epigenetic, and metabolic characteristics of muscle tissue [34], potentially leading to different outcomes in response to mechanical tension. 

Cellular gene expression and tissue formation are inherently dynamic processes. C2C12 cells within 3D scaffolds display maturation profiles that are dependent on time, making the timing of external stimuli application crucial owing to the potential for varying myogenic outcomes at different tissue maturation stages [23]. 

In this study, uniaxial static strain was applied to cell-seeded, collagen-coated PCL scaffolds at two onset times: an early time point at 72 h and a delayed time point at 120 h. During mechanical testing, the stress values remained within the elastic range of the sample [25]. For tissue engineering applications, it is imperative to maintain asepsis and a sterile environment [35], ensure appropriate temperature and environmental parameters, and utilize a proper loading transmission system [24]. When conducting such assays, it is crucial to keep these conditions at optimal levels. 

Application of static strain at 72 h resulted in a slight decrease in cell proliferation, whereas control cells exhibited a significant increase in proliferation. In response to suitable environmental cues, proliferating myoblasts exit the cell cycle to commit to the myogenic lineage and undergo differentiation. Cell cycle arrest occurs early in the myogenesis process and is crucial for the development of the contractile phenotype and cellular aging [36,37]. The slight decrease in cell proliferation present in the mechanically stimulated scaffolds holds promise as an indicator of cell cycle arrest. Withdrawal from the cell cycle is facilitated by the expression of MyoD, which interacts with cell cycle regulators and inhibits cytokine signaling [9,38]. According to Walsh and Perlman, this event is essential as the progression of the cell cycle inhibits differentiation [36]. Results from the experiments are consistent with these findings (see Figure 4a). 

Fluorescent staining revealed that cells not only remained attached to the scaffold post-strain application, but also responded to the mechanical cues initiated by the anchoring and stretching of the scaffold. Mechanical strain promoted the elongation and reorientation of cell nuclei, aligning them with the direction of the microfilaments and the strain (see Figure 4c). The application of mechanical forces induces substantial structural modification within the cytoplasm and the nucleus. Elongated cell morphology arises from the generation of increased tension, either as a byproduct of contractile forces produced during process of myofibril formation, or, alternatively, during nuclear translocation facilitated by the microtubule and/or microfilament systems [39]. 

Cells typically orient themselves based on substrate topography; however, mechanical strain is also known to promote cell elongation and alignment through the activation of mechanotransduction pathways [23,39]. For instance, pulling on integrins results in the reorganization of individual actin stress fibers and the nucleus, leading them to shift their position and align with the newly established tension field lines [28]. A difference in the alignment of the nuclei was observed when the strained and control samples were contrasted (refer to Figure S3). The alignment and elongation observed in the nuclei of the control sample is attributed to both the scaffold’s topography and tension generated exclusively by the anchoring of the microfilaments to the grip system’s knobs.

Aligned cells are more likely to fuse in a parallel manner, resulting in the formation of myotubes that are structurally organized and exhibit properties characteristic of native muscle tissue. This structural organization enhances the contractile function of the myotubes, as the uniform alignment allows for more efficient force generation along the length of the muscle fibers. Therefore, the observed cell elongation and alignment in response to uniaxial static tension not only contribute to the structural integrity of the engineered muscle tissue, but also enhance its mechanical functional capabilities [40], bringing the in vitro constructs closer to mimicking the properties of native muscle. 

Previous studies have demonstrated that static strain applied to cell-seeded scaffolds promotes alignment, which is specifically important for facilitating the expression of myogenic genes, fusion, directed myotube formation, and maturation [20,23]. For instance, Heher et al. applied 10% static strain for 6 h and 3% static strain for 18 h to myoblasts embedded in fibrin matrices, resulting in elongated cells which oriented themselves in the direction of the strain and nuclei transitioning from round to stretched shapes [20]. The loading pattern applied in the present study was selected following Heher et al. and similar results were obtained. 

Alongside morphological changes, alterations in cell expression were noted. The MyoD myogenic marker was detected in the cytoplasm of the strained cell-seeded scaffold. While MyoD presence is typically noted within the nucleus [9], instances of cytoplasmic MyoD-positive cells have been documented and are associated with early stages of differentiation (refer to Figure 4d). Yamamoto et al. reported a heterogeneous population of MyoD-positive cells, with some showing nuclear expression and others showing cytoplasmic expression after one day of culture on a 2D substrate, with both groups participating in the fusion process [39]. As depicted in Figure 4d, the expression of MyoD on the strained scaffold resembles the cytoplasmic MyoD identified by Yamamoto et al. MyoD presence coincides with the suppression of cell proliferation previously recorded. 

Myogenin expression typically precedes terminal differentiation, with peak expression observed during the middle stages of myogenic development [9]. Given that Myogenin was not detected in either the strained or control samples, while MyoD was only present in the strained scaffold, it can be inferred that mechanical stimulation initiated at 72 h of culture promoted an early stage of myogenic differentiation.

Previous research has shown that delayed application of mechanical strain can enhance myogenic phenotypic outcomes [23]. Accordingly, mechanical stimulation was applied to the cell-seeded scaffold after 120 h of static cell culture (5 days) with the goal of promoting a phenotypic outcome. Cell proliferation, expression of myogenic markers MyoD and Myogenin, and cell alignment were then evaluated. Following mechanical strain, cell proliferation was suppressed, suggesting an exit from the cell cycle—a strong indicator of commitment to the myogenic lineage and initiation of differentiation [36]. 

When mechanical stimulation was initiated at 120 h, the activity of MyoD not only promoted cell-cycle exit but also triggered Myogenin expression, supporting the existence of a positive autoregulatory loop between these myogenic regulatory factors [9]. Unlike MyoD, Myogenin was primarily localized within the nucleus of cells, though it was also observed in the cytoplasm. According to Ferri et al., Myogenin is found in the cytoplasm of undifferentiated cells but translocates to the nucleus upon differentiation induction when proliferation signals cease [9].

The presence of Myogenin-positive nuclei in the samples post-cell cycle exit suggests that a population of cells is at the middle stages of myogenesis, actively differentiating. Myogenin plays a pivotal role in orchestrating differentiation through myocyte fusion, myotube formation, and synthesis of contractile proteins [8,9]. The identification of fusion and, consequently, multinucleated muscle cells or myotubes, was exclusively observed in the strained scaffold (refer to Figure 7). Analyzing myosin heavy chain (MHC) expression provides valuable insights into myotube orientation. Therefore, assessing its expression is recommended to better understand and evaluate the development of myotube structure [41]. 

While the study demonstrates significant morphological and molecular changes in myoblast differentiation under mechanical stimulation, the specific signaling pathways driving these changes have not been explicitly characterized. Mechanical stimulation is known to influence muscle cell growth, differentiation, and morphology through various intracellular molecular pathways [42] including PI3K/Akt/mTOR [43,44] MAPK/ERK [43,45], and Wnt/β-catenin [10,38], which were not evaluated in this study. This represents a limitation of the current work. Future studies should aim to assess gene expression and protein quantification related to these pathways to better understand the mechanisms underlying myogenic differentiation. 

Furthermore, uniaxial alignment after mechanical stimulation was observed. Both strained and control scaffolds exhibited cell alignment due to passive tension induced by the arrangement of the microfilaments in the grip systems over a 144 h period. However, the strained scaffold showed a higher percentage of nuclei oriented within a −5° to 5° tilt angle, although the differences were not statistically significant. Given the high variability observed in 3D scaffolds, a larger sample size may be required in future experiments to achieve statistical significance in the data. In addition, resources for immunofluorescence analysis were limited; thus, assessments for the expression of myogenic markers and cell alignment were only performed following stimulation. Future studies should also study cell gene expression and morphology before mechanical stimulation to provide a more comprehensive understanding of the baseline cellular conditions, allowing for a more accurate interpretation of the effects induced by mechanical stimulation. 

Different onset times for mechanical stimulation have been reported in the literature. For instance, Candiani et al. maintained a static culture of C2C12 cells in a microfibrous membrane for 5 days before subjecting the cells to strain, Heher et al. applied mechanical stimulation to their C2C12 cell-laden fibrin matrix at an onset time of 3 days, and Moon et al. cultured primary human muscle precursor cells in a collagen-based matrix for 2 days before applying cyclic stretch. These authors reported positive responses to mechanical stimulation such as MHC accumulation, cell alignment, myotube formation, and expression of myogenic markers [20,29,31]. Nevertheless, it is important to consider that different cells and scaffolds were utilized in these studies; therefore, protocol optimization is crucial in each specific case.

Based on the proliferative rate of C2C12 cells in the collagen-coated PCL scaffold previously documented in the present research, a minimum duration of 72 h (3 days) of static culture was deemed necessary to facilitate an adequate cell density on the surface of the scaffold to withstand mechanical stimulation effectively. Conversely, at 168 h (7 days) of culture, the confluence on the scaffold was high, which could cause premature differentiation. 

Considering this analysis, an onset time of 72 h was first tested. As evidenced by the results, the loading pattern applied to the cell-seeded scaffold was effective in promoting an early stage of differentiation, characterized by MyoD expression and a lack of myotube formation. To further promote myogenic outcomes, an onset time of 120 h was then assessed, using the same loading pattern and duration. This variable within the protocol demonstrated efficacy, as co-expression of MyoD and Myogenin was observed, and multinucleated cells were observed, suggesting that the cells were in the middle stage of myogenesis. These observations align closely with the findings of Somers et al., who investigated the effects of initiating strain at both 72 h and 120 h in their C2C12 cell-laden fibrin scaffold. Their study concluded that delaying the application of strain until a more mature stage of cellular development led to more favorable myogenic outcomes, such as the formation of larger myotubes and the presence of visible striations [23]. 

Several factors may have contributed to the improved outcomes observed when mechanical strain was applied at 120 h. Initially, the cells experienced a prolonged period of passive tension due to the anchoring of microfilaments to the grip system. This allowed them to acclimate to the topographical and mechanical cues of the scaffold over an extended duration. Moreover, the extended static culture phase prior to strain application may have prolonged the cell proliferation stage, resulting in increased cell density. This denser cellular environment likely facilitated enhanced cell–cell contact, which is crucial for appropriate cell differentiation.

## 5. Conclusions

The present study demonstrated that the employed mechanical stimulation assay effectively promotes myoblast differentiation in cell-seeded collagen-coated PCL microfilament scaffolds. The application of uniaxial static strain induced significant morphological and molecular changes, which are evidence of the differentiation process. Importantly, the results underscore the critical role of myoblast maturity at the time of strain application in determining myogenic outcomes. Mechanical stimulation initiated at 72 h facilitated an early stage of differentiation, predominantly characterized by MyoD expression. Conversely, stimulation applied at 120 h supported progression to a middle differentiation stage, where both MyoD and Myogenin were co-expressed, culminating in the formation of myotubes.

These results emphasize the importance of time in the application of mechanical cues in tissue engineering strategies to optimize myogenic differentiation. The observed differential expression of myogenic markers and subsequent myotube formation at varied onset times of mechanical stimulation suggests a nuanced interaction between mechanical forces and cellular maturation processes. This interaction warrants further investigation to elucidate the underlying mechanisms and to refine protocols for enhanced muscle tissue engineering outcomes.

## 6. Patents

A patent for the biomechanical stimulation system is currently under evaluation. No 278368 (IN2021563599), with classification C12N 58, A6L 27/38.

## Figures and Tables

**Table 1:** Experimental timeline of mechanical stimulation tests.

Time (h)	Onset Time of Stimulation 72 h	Onset Time of Stimulation 120 h
0	Cell seeding on scaffold.	Cell seeding on scaffold.
24	Static culture (horizontal position).	Static culture (horizontal position).
48		
72	Cell proliferation measurement. Coupling of biomechanical stimulation system. Mechanical stimulation.	
96	End of mechanical stimulation. Cell proliferation measurement. Immunostaining.	
120	-	Cell proliferation measurement. Coupling of biomechanical stimulation system. Mechanical stimulation.
144	-	End of mechanical stimulation. Cell proliferation measurement. Immunostaining.

## Data Availability

The data that support the findings of this research are available upon reasonable request from the corresponding author.

## Supplementary Materials

The following supporting information can be downloaded at: https://www.mdpi.com/article/10.3390/bioengineering11090919/s1, Figure S1: Immunofluorescent staining of cell-seeded scaffold after 24 h of mechanical stimulation. Onset time of mechanical stimulation 72 h. (a) Expression of MyoD myogenic marker. (b) Expression of Myogenin myogenic marker. DAPI (blue), Phalloidin (green) and MyoD/Myogenin (red). Merge shows the overlay of DAPI, Phalloidin and MyoD/Myogenin (blue, green and red) (20×); Figure S2: Directionality histograms of strained and control cell-seeded scaffolds. Onset time of mechanical stimulation 72 h and 120 h. No significant differences were found between the data (*p* ≥ 0.05); Figure S3: Immunofluorescent staining of cell-seeded scaffold after 24 h of mechanical stimulation. Onset time of mechanical stimulation 120 h. (a) Expression of MyoD myogenic marker. (b) Expression of Myogenin myogenic marker. DAPI (blue), Phalloidin (green) and MyoD/Myogenin (red). Merge shows the overlay of DAPI, Phalloidin and MyoD/Myogenin (blue, green and red) (20×).
