# Supplementary material for: Impact of Uniaxial Static Strain on Myoblast Differentiation in Collagen-Coated PCL Microfilament Scaffolds: Role of Onset Time of Mechanical Stimulation
Source: Bioengineering (Basel). 2024 Sep 13;11(9):919. doi: 10.3390/bioengineering11090919 (PMC11428666; doi:10.3390/bioengineering11090919)
Supplement: Supplementary file 1 [file bioengineering-11-00919-s001.zip › bioengineering-3182035-supplementary.pdf]

bioengineering

Supplementary Materials

Impact of Uniaxial Static Strain on Myoblast Differentiation in Collagen-Coated PCL Microfilament Scaffolds: Role of Onset Time of Mechanical Stimulation

María Laura Espinoza-Álvarez [1,2,*], Laura Rojas-Rojas [1,3], Johan Morales-Sánchez [2,4] and Teodolito Guillén-Girón [1]

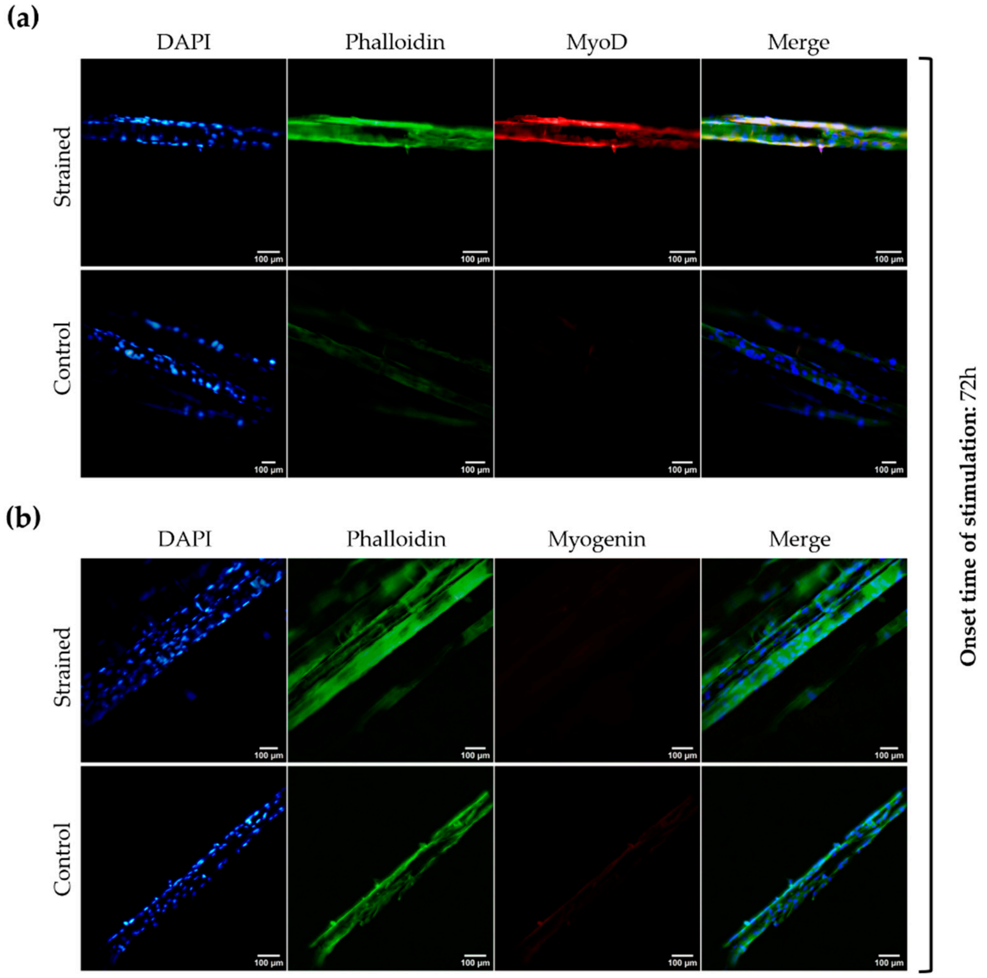

Figure S1. Immunofluorescent staining of cell-seeded scaffold after 24 h of mechanical stimulation. Onset time of mechanical stimulation 72 h. (a) Expression of MyoD myogenic marker. (b) Expression of Myogenin myogenic marker. DAPI (blue), Phalloidin (green) and MyoD/Myogenin (red). Merge shows the overlay of DAPI, Phalloidin and MyoD/Myogenin (blue, green and red) (20x).

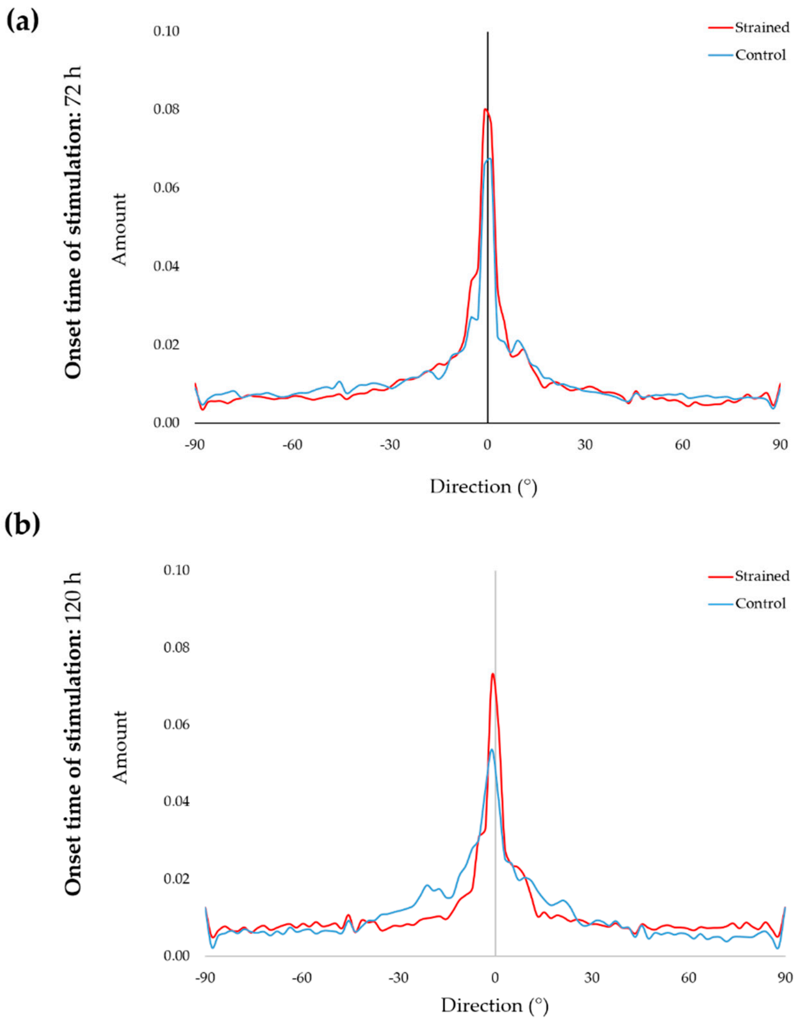

Figure S2. Directionality histograms of strained and control cell-seeded scaffolds. Onset time of mechanical stimulation 72 h and 120 h. *No significant differences were found between the data* ($p \geq 0.05$).

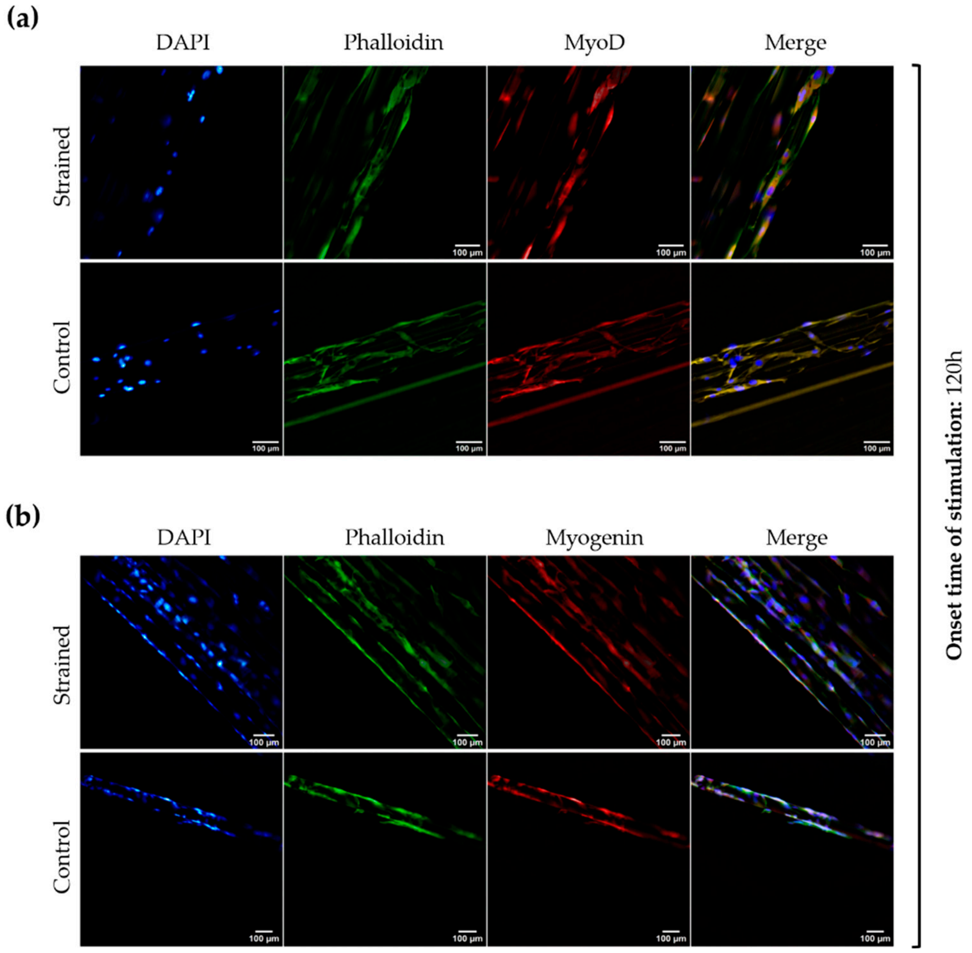

Figure S3. Immunofluorescent staining of cell-seeded scaffold after 24 h of mechanical stimulation. Onset time of mechanical stimulation 120 h. (a) Expression of MyoD myogenic marker. (b) Expression of Myogenin myogenic marker. DAPI (blue), Phalloidin (green) and MyoD/Myogenin (red). Merge shows the overlay of DAPI, Phalloidin and MyoD/Myogenin (blue, green and red) (20x).
